# Treatment of head lice with dimeticone 4% lotion: comparison of two formulations in a randomised controlled trial in rural Turkey

**DOI:** 10.1186/1471-2458-9-441

**Published:** 2009-12-01

**Authors:** Özgür Kurt, I Cüneyt Balcıoğlu, Ian F Burgess, M Emin Limoncu, Nogay Girginkardeşler, Tuba Tabak, Hasan Muslu, Özge Ermiş, M Turhan Sahin, Cemal Bilac, Hakan Kavur, Yusuf Özbel

**Affiliations:** 1 Celal Bayar University Medical School, Department of Parasitology, Manisa, Turkey; 2 Medical Entomology Centre, Cambridge, UK; 3 Celal Bayar University Medical School, Vocational School of Health Services, Manisa, Turkey; 4 Celal Bayar University Medical School, Department of Dermatology, Manisa, Turkey; 5 Ege University Medical School, Department of Parasitology, Bornova, Izmir, Turkey

## Abstract

**Background:**

Dimeticone 4% lotion was shown to be an effective treatment for head louse infestation in two randomised controlled trials in England. It is not affected by insecticide resistance but efficacy obtained (70-75%) was lower than expected. This study was designed to evaluate efficacy of dimeticone 4% lotion in a geographically, socially, and culturally different setting, in rural Turkey and, in order to achieve blinding, it was compared with a potential alternative formulation.

**Methods:**

Children from two village schools were screened for head lice by detection combing. All infested students and family members could participate, giving access to treatment for the whole community. Two investigator applied treatments were given 7 days apart. Outcome was assessed by detection combing three times between treatments and twice the week following second treatment.

**Results:**

In the intention to treat group 35/36 treated using dimeticone 4% had no lice after the second treatment but there were two protocol violators giving 91.7% treatment success. The alternative product gave 30/36 (83.3%) treatment success, a difference of 8.4% (95% CI -9.8% to 26.2%). The cure rates per-protocol were 33/34 (97.1%) and 30/35 (85.7%) respectively. We were unable to find any newly emerged louse nymphs on 77.8% of dimeticone 4% treated participants or on 66.7% of those treated with the alternative formulation. No adverse events were identified.

**Conclusion:**

Our results confirm the efficacy of dimeticone 4% lotion against lice and eggs and we found no detectable difference between this product and dimeticone 4% lotion with nerolidol 2% added. We believe that the high cure rate was related to the lower intensity of infestation in Turkey, together with the level of community engagement, compared with previous studies in the UK.

**Trial Registration:**

Current Controlled Trials ISRCTN10431107

## Background

It is standard practice, and ethically sound, for randomised, controlled trials of new treatments to be conducted in the geographic territory where the product will eventually be used. In the case of treatments for head lice there is an additional reason that competent authorities require data from their home territories. In most developed countries prior use of insecticides has selected populations of lice that have acquired resistance to one or more active substances, mostly insecticides, so trials conducted in a country or region where insecticides have not been used extensively, or even at all, may show an outcome that would not accurately reflect the likely outcome in a country where insecticides are widely available.

Clinical trials in the United Kingdom (UK) necessarily rely upon volunteer participants who may be scattered throughout a community. This means that those individuals may be effectively treated but, because of the close nature of much of childhood play, the children taking part in the studies may come into close contact with others who have head lice, with the result they become reinfested either during the trial or soon after its completion. Reinfestation within the family may occur readily in normal circumstances but, because all household members either participate or are treated in UK clinical studies, reinfestation within the study period can often be identified as coming from outside contacts. With this prospect in mind our previous clinical studies conducted in the UK have always included a provision for recognising reinfestation during the course of a trial, by means of a selective algorithm based upon the number of lice found and the developmental stage of those lice [1-3]. However, it is not selective enough to be able to identify reinfestation at intensity greater than permitted by the algorithm.

In contrast with the UK, clinical trials conducted in developing countries often engage whole communities in the process of therapy. This has a number of advantages. For the community it provides access for all members of the community to that aspect of healthcare, irrespective of their socio-economic status, reducing the risk of ongoing transmission of an infectious agent, as well as reducing the level of morbidity resulting from that infection. This kind of study has been reasonably successful for control of lice and other infestations when a study cohort comprises a whole community on a single intervention [4,5]. We have previously worked with several village communities in Manisa province, Turkey, on a variety of internal and external parasites [6-9]. The villagers appreciate the health input and there is a high level of participation in each investigation.

Turkey has a plethora of lotions, crème rinses, and shampoos based on permethrin or d-phenothrin but no treatment or product that, by virtue of its dominant position in the market or outstanding effectiveness, could be considered a "gold standard" to be used for comparison with new products in any clinical study conducted there. Dimeticone 4% lotion has recently (spring 2008) been approved by the Turkish competent authority for sale to the public. This study enabled the product to be evaluated for efficacy in a socially and geographically different population from those involved in previous investigations. It also evaluated the efficacy of the dimeticone 4% lotion in comparison with an alternative dimeticone 4% lotion product containing 2% nerolidol as a surface tension modifier, which was being considered as an alternative to the original product based on *in vitro *data, in a randomised, controlled, assessor blind, parallel group, community study.

## Methods

### Objectives

The study was designed to evaluate the efficacy of dimeticone 4% lotion against head louse infestation in a different study population from previously. It was also designed to compare dimeticone 4% lotion with a potential alternative silicone formulation. It was anticipated that for both objectives the risk of reinfestation would be minimised, compared with previous investigations, by offering treatment to the whole community.

### Participants

The study was based around two rural village schools in Manisa province, western Turkey. The villages were selected because the schools were large enough to provide a sufficient number of participants but small enough for the investigation team to screen all students for head lice during a single day. One school in the village of Osmancali serves approximately 20 small villages and communities in the locality and children are bussed in daily. The other school in Maldan village serves only that community and a few adjacent farms. Children in each of the schools were screened for presence of live lice using plastic detection combs ("PDC", KSL Consulting, Denmark) [6]. Infested children were provided with an information sheet to take home and subsequently the family of every child found positive for head lice was visited at home by at least one investigator and the community nurse. A standard consent and assent procedure was followed. All infested family members were able to participate in the study.

All enrolled participants provided baseline data on age, gender, hair characteristics, and previous pediculicide use. The lower age limit was 4 years; there was no upper limit. Treatments and assessments were conducted in the school, which ensured access to all participants. When children were not available for assessment on the assigned school day a follow up was arranged by the locality nurse to visit them at home. No payment was offered for participation.

Inclusion and exclusion criteria for this study were essentially the same as we have used previously [1-3], excluding potential participants with: known sensitivity to any component of treatment; secondary bacterial infection or other chronic scalp condition; bleached, coloured, or permanently waved hair within the previous four weeks; use of pediculicide within two weeks or co-trimoxazole or trimethoprim antibiotics within four weeks (either of which could affect the viability of any lice remaining on the head). Pregnancy and breast feeding were also exclusion factors, as were having participated in another clinical study within 1 month or prior participation in this study.

### Ethics

The study was granted ethical approval by the ethics committee of the Medical School of Celal Bayar University, Manisa, Turkey (Study 025, 14/02/2007). Approval was also obtained from the Board of Education covering that part of Manisa province as well as individual school authorities prior to commencement. None of the children attending the two schools was older than 14 years of age so parents signed a form giving written consent for them to participate and stating they understood the purpose of the study as set out in the information leaflet. All the children also gave written assent prior to enrolment.

The study was conducted in conformity with the principles of the Declaration of Helsinki and of European Directive 2001/20/EC.

### Treatment

Two physically similar silicone lotions were used for this study. Both were based on 4% dimeticone in a cylcomethicone solvent. The original dimeticone 4% lotion (Hedrin^® ^4% lotion, Thornton & Ross Ltd, Huddersfield, UK) was compared with essentially the same mixture into which 2% nerolidol had been incorporated. Nerolidol is a linear sesquiterpene alcohol that has properties that may allow it to modify the surface tension of the lotion and facilitate its penetration into the breathing apparatus of the louse and its egg. Tests *in vitro *indicted that it had greater ability to enter the breathing system of the louse eggs, resulting in reduced risk of hatching by a factor of about 30%.

Participants were randomised to receive either dimeticone 4% lotion or dimeticone 4% lotion with nerolidol 2%, each with two applications 7 days apart. As in previous studies of dimeticone 4% lotion the products were applied by members of the investigation team. Sufficient product was applied to dry hair to moisten the whole scalp and length of the hair. Treatment was applied at the end of the school day, left to dry naturally, and washed off the following morning at home, using a non-medicated shampoo supplied by the investigation team. Family members who were found with lice but were unable to participate for any reason were provided with dimeticone 4% lotion for self administration in order to reduce the risk of reinfestation to the study group. Only sixteen people reported having ever used a pediculicide, six had used permethrin about 2 months previously with varying results; the others had not been treated for between 6 months and 6 years.

### Assessment of Outcome

At initial diagnosis an assessment of intensity of infestation was based on the rapidity with which lice were found during the initial combing. This measure was used previously [1-3] as a guideline during post-study evaluations of outcome: heavy = >1 louse with the first stroke of the comb, medium = 1 louse with the first stroke of the comb, light = 1 louse found only after 5-6 strokes of the comb.

Each participant was assessed for presence of lice on five occasions, days 1, 2, 6, 9, and 14 after the first application of treatment, using the "PDC" comb on dry hair in the same way as the children were initially screened in the schools. The purpose of the additional combing immediately following the first treatment (i.e. a combing on days 1 and 2 rather than on day 1 or day 2 as have been used in other studies) was to enable the investigators a better opportunity to find any newly hatched nymphs in relation to evaluation of the first primary outcome measure, the ovicidal effect of the treatments, defined as no nymphal lice emerging from eggs between treatments. the other primary outcome measure, elimination of live lice following the second application of treatment, that is, no lice present on days 9 and 14, was monitored by combing on days 9 and 14 as in previous studies [1-3].

Interim combing assessments employed the same approach as previously, drawing the comb 2-3 times through each section of hair. This level of dry combing has minimal intervention effect because, if no lice are found the combing cannot affect the outcome of treatment in any way. If lice are found the non-intensive combing removes only a proportion of any lice present, but allows identification of treatment failure or possible reinfestation at the earliest opportunity. The day 14 assessment involved more extensive combing than on other days to ensure no live lice were present. Non-participating family members with lice who self treated were also checked if they assented. This allowed us to confirm whether their treatments had been successful and to understand the circumstances that could lead to reinfestation. Reinfestation was defined using an algorithm described previously [2,3].

### Sample size and Randomisation

Previous studies conducted in the UK have demonstrated efficacy rates for dimeticone 4% lotion of around 70% and no newly emerged nymphs have been found on approximately 50% of participants after first treatment [1,3]. Based on group sizes used previously a sample size of 31 per group was considered to have at least 80% power to detect (with 95% confidence) a difference of 35% between the products in not finding newly hatched nymphs or in efficacy rates based on elimination of infestation. The actual sample sizes of 36 per group made allowance for dropout. Treatment allocation was in balanced blocks of 12 predetermined using a computer generated list (http://www.randomization.com, 17 March 2008). Allocation at the point of delivery was made from instruction sheets enclosed in opaque, sealed, sequentially numbered envelopes distributed to the investigators in batches of 12. As each participant was enrolled the investigator on site selected the next available numbered envelope from the allocation and the treatment was then applied from one of two series of numbered bottles.

Randomisation was by individual so if more than one member of a family was enrolled it was possible for them to receive different treatments. This approach for studies on head louse infestation has been the accepted randomisation methodology by the Medicines and Healthcare products Regulatory Agency (MHRA) in the UK, whereas the US Food and Drug Administration (FDA) have been inclined towards cluster randomisation by family. However, the latter methodology is generally not practical for a small proof of concept study as there is a need to stratify households according to number of participants, and possibly even intensity of infestation of individuals within the households, and the working assumption is generally that all the household units are independent and the participants within a household are correlated. A statistical difficulty arises on how to deal with within-household correlation because some of the evidence (unpublished) from previous studies indicates that for some households there is no within-household correlation. Consequently, it was decided at an early stage to continue to use randomisation by individual.

As the two products were physically similar the allocation was essentially blinded to participants at the point of delivery. In order to avoid contamination bias all study treatments were applied by investigators and the bottles of study medication returned to the centre for weighing after single use. Assessments were performed by different investigators from those who conducted the initial screening, enrolment, and treatment of each child in order to reduce the possibility of bias.

### Statistical methods

For presence/absence variables a Fisher exact test was used. Differences in success rates between the treatment outcomes for the two preparations were quantified by the 95% confidence interval, calculated using a normal approximation to the binomial distribution. For graded or semi-continuous variables, Kruskal-Wallis analysis of variance was used, which as only two groups were tested was equivalent to using the Mann-Whitney U test.

## Results

### Recruitment and participant flow

**Figure 1:**
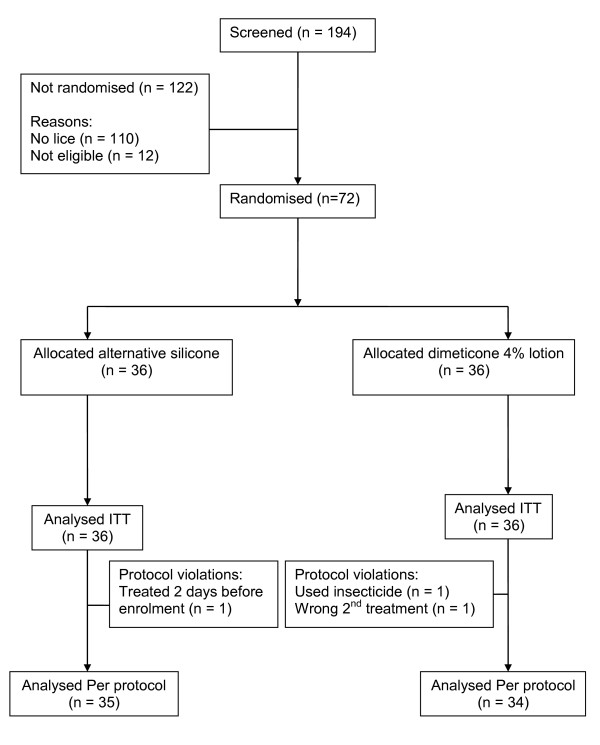
**Flowchart of subjects in study**.

The study was conducted throughout April and May 2008. During this time 72 participants from 57 families distributed between 12 villages were recruited to receive either dimeticone 4% lotion or the alternative silicone preparation (Figure 1). Nineteen participants came from Osmancali village and 34 from Maldan, but no more than four came from any one of the other ten villages involved, each of which was served by Osmancali School. Thirteen families had two participants in the study and one family three participants. Where multiple family members were included in the analysis, five families, with all members treated using the same product, occurred in each of the treatment groups. Only four families had members in different treatment groups. Sixty nine participants completed the study as required by the protocol.

### Baseline data

Demographic characteristics of the study population at baseline are given in Table 1. Overall the groups were similar with no significant differences. However, in this study there was a non-significant trend for a greater proportion of children with fine hair and light infestation compared with previous studies conducted in the UK [1,3]. Both these factors are to some extent subjective to evaluate and could be influenced by the hair characteristics and the relative experience of the investigator.

**Table 1:** Comparison of demographic characteristics of participants

	Dimeticone 4% lotion	Dimeticone + nerolidol lotion	
	(n = 36)	(n = 36)	
Age in years:			
range	6 to 31	5 to 37	
mean	11.4	11.6	
median	10	10	
	number of participants (%)	number of participants (%)	p value
			
Gender:			
male	9 (25.0)	7 (19.4)	0.777
female	27 (75.0)	29 (80.6)	
			
Infestation:			
light	30 (83.3)	27 (75.0)	0.670
medium	3 (8.3)	5 (13.9)	
heavy	3 (8.3)	4 (11.1)	
			
Hair length:			
close cut	6 (16.7)	5 (13.9)	0.925
above ears	3 (8.3)	2 (5.6)	
ears to shoulders	10 (27.8)	12 (33.3)	
below shoulders	17 (47.2)	17 (47.2)	
			
Hair thickness:			
fine	17 (47.2)	14 (38.9)	0.869
average	16 (44.4)	14 (38.9)	
thick	7 (19.4)	8 (22.2)	
			
Hair curl:			
straight	17 (47.2)	25 (69.4)	0.115
wavy	15 (41.7)	10 (27.8)	
curly	4 (11.1)	1 (2.8)	
			
Hair oiliness:			
dry	7 (19.4)	9 (25.0)	0.813
normal	25 (69.4)	24 (66.7)	
oily	4 (11.1)	3 (8.3)	

### Outcomes

Seventy two participants were enrolled in the study. Of these 35/36 (97.2%) of the dimeticone 4% group were free from lice on both assessment days (days 9 and 14) following the second treatment on day 7. However, two people were subsequently excluded from per-protocol evaluations, one because it was found they had been treated with a non-study pediculicide product during the study and the other because they had used a treatment only 2 days prior to enrolment but had not informed investigators. In the group treated with the dimeticone and nerolidol preparation, 31/36 participants (86.1%) were found to have no lice at both assessments after the second treatment but one person was excluded from per-protocol evaluation because they used a non-study product during the study. This resulted in a worst case analysis outcome of 33/36 (91.7%) successful treatments for dimeticone 4% lotion and 30/36 (83.3%), one of which was considered cured but subsequently reinfested, for the alternative dimeticone 4% preparation with 2% nerolidol added, with protocol violators included nominally as treatment failures. The difference, 8.4%, has a probability of 0.478 with a 95% confidence interval of -9.8 to 26.2 and indicates no difference of activity between the products, but the numbers involved in the study were too small to be conclusive. All compliant participants had complete data sets so the per-protocol outcome was 33/34 (97.1%) and 30/35 (85.7%) respectively with successful treatments. This provides a difference of 11.4%, which has a probability of 0.198 with a confidence interval of -5.3 to 28.3, which also indicates no significant difference between the treatments.

A sub-group analysis based on demographic characteristics showed no difference between the groups in the rate of cure or re-infestation in any of the subgroups analysed apart from those with "Normal" hair for whom dimeticone 4% lotion was significantly (p < 0.05) more effective (Table 2). However, this difference can be considered a "false positive" effect as, based on previous experience, there is no a priori reason to expect dimeticone 4% to be more effective when used on "Normal" hair.

**Table 2:** Success of treatment by sub-group (ITT population)

	Dimeticone 4% lotion		Dimeticone + nerolidol lotion		
Sub-group	n/N	%	n/N	%	p
All subjects	33/34	97.1	30/35	85.7	0.198
					
Sex:					
males	9/9	100.0	7/7	100.0	*
females	24/27	88.9	23/28	82.1	0.705
					
Age:					
5 to 7	5/5	100.0	3/5	60.0	0.444
8 to 10	14/14	100.0	16/19	84.2	0.244
11 to 16	12/13	92.3	8/8	100.0	*
17+	2/2	100.0	2/2	100.0	*
					
Infection:					
light	29/29	100.0	24/26	92.3	0.219
moderate	2/3	66.7	2/5	40.0	*
heavy	3/3	100.0	3/3	100.0	*
					
Hair length:					
close cut	6/6	100.0	5/5	100.0	*
above ears	3/3	100.0	2/2	100.0	*
ears to shoulders	9/10	90.0	10/11	90.9	*
below shoulders	15/15	100.0	13/17	76.5	0.104
					
Hair thickness:					
fine	10/11	90.9	10/13	76.9	0.596
medium	16/16	100.0	12/14	85.7	0.209
Thick	7/7	100.0	8/8	100.0	*
					
Hair curl:					
straight	15/16	93.8	21/24	87.5	0.638
wavy or curly	18/18	100.0	9/11	81.8	0.136
					
Hair type:					
normal	23/23	100.0	18/23	78.3	0.049
other	10/11	90.9	12/12	100.0	0.478
					
Other family member in study:					
no	18/18	100.0	19/21	90.5	0.490
yes	15/16	93.8	11/14	78.6	0.316

*where no p value is specified, p = 1.0

Analysis of data collected at interim assessments on days 1, 2, and 6 found recently hatched nymphs on 8 participants treated using dimeticone 4% and 11 participants treated with the alternative product, indicating that inhibition of nymphs hatching after the first treatment, was 28/36 (77.8%) and 24/36 (66.7%) respectively (p = 0.85, 95% CI, -10% to 32%). In seven of the cases who had nymphs following the first treatment with dimeticone 4%, no lice were found after the second treatment on day 7.

Amongst the subjects with lice present on days 1, 2, or 6, there were five groups of siblings. In at least two families, each with two participating members, another family member was found to be infested but not enrolled. It is not known whether those non-participants were treated adequately, or even at all, at the commencement of the study and could have constituted a source of reinfestation. However, in the four families with a participating member in each treatment group there were no treatment failures. Of the families in which all members were treated using the same product, four families treated with dimeticone 4% were all cleared of lice and one family had one successful and one failed treatment; three families treated using dimeticone 4% with nerolidol were all cured, one family had the treatments of both participants fail, and the family with three members had one success, one failure, and one appeared to have been reinfested after having lice eliminated.

Two of the treatment failures were siblings from Osmancali the others were all from Maldan village. No two treatment failures were the same age so there were no obvious correlating factors, such as participants with lice being playmates, or other detectable differences in overall efficacy between the schools involved.

### Adverse events

No adverse events due to treatment or unrelated illnesses were reported by participants in this study. There were also no changes in concomitant medication.

## Discussion

We have conducted a randomised, assessor blinded comparison between dimeticone 4% lotion and dimeticone 4% lotion with nerolidol 2% added. From our *in vitro *work we had anticipated that using the formulation with nerolidol could improve treatment outcome by exhibiting a greater effect to inhibit hatching of louse eggs following the first application of treatment. However, in this study any difference of activity against louse eggs was either not apparent or else not detectable due to the overall high proportion of successful treatments using both products. Despite the nerolidol formulation not performing as well as anticipated, based on *in vitro *data, there was also no evidence that addition of nerolidol to dimeticone 4% lotion diminished its activity in any way.

Previously it was found that, although dimeticone 4% lotion was effective to eliminate head louse infestation, a proportion of participants in studies conducted in the UK were not cured according to study criteria or else had been reinfested to such a degree from contacts in the community that the treatment was considered to have failed [1,3]. Part of the reason for this is that in recent years the prevalence of infestation in the UK has probably been higher than at any time since the 1940s and many individual cases show intense infestation with hundreds of lice present. However, in this study we have found that when dimeticone lotions were used in a setting where the overall intensity of infestation was lower and all members of a community diagnosed with head louse infestation had access to treatment concurrently, it was possible to reduce the risk of rapid reinfestation from contacts during the course of the study.

Comparison of the data obtained in Turkey with those from the UK [3] showed that dimeticone 4% gave a significantly more effective elimination of infestation using worst case intention to treat analysis, in which 33/36 (91.7%) participants were free from lice after the second treatment compared with 30/43 (69.8%) in the UK study, a difference of 21.9% (p < 0.015, confidence interval 4% to 40%). Per protocol outcomes of 33/34 and 30/39 respectively showed a similar difference (p < 0.013, confidence interval 4% to 36%).

We found the success rate using silicone treatments in this study was significantly better at all levels than we were able to achieve in the UK [3]. Most demographic differences were non-significant apart from the level of intensity of infestation and the coverage of treatment within the community. This difference highlights the potential difference of outcome likely to be encountered in conducting studies in different countries and reinforces the preference of some Western European competent authorities for conducting studies on "home soil" rather than in third party countries. However, there are other factors that influence outcome, for example in the UK study we relied on volunteer participants who came from different places and, although some of them did respond to advertising in clusters, the overall coverage of each village community was sparse. This meant that prior to recruitment there was a greater chance respondents had longer term and more intense infestations in the UK, where light infestations were found in only 14/43 (32.6%) participants versus 57/72 (79.2%) in Turkey (p < 0.0004, confidence interval 28% to 65%), and after treatment more potentially infective contacts for study participants remained untreated, with the result that the risk of reinfestation was higher in the UK. In this study we screened all school aged children in the two communities, which should have identified individuals with the highest risk of infestations, and through them family members also with a high risk. By eliminating lice from the highest risk group of families, the overall risk of reinfestation from those communities should have been reduced. However, if individuals outside the school groups declined screening or treatment, or if families with potentially infested younger children did not come forward for screening, a risk for reinfestation remained for any participants with whom they had contact.

One concern raised during earlier investigations, and following consumer use of silicone lotions containing dimeticone, was that perhaps some lice, or louse infestations, are more difficult to eliminate than others. As resistance is not considered a likely occurrence with dimeticone treatment due to its physical mode of action, it was asked whether some lice had innate potential to avoid or tolerate exposure to the silicone fluid compared with other lice. It appears that what we observed was not a failure to kill lice but rather an inability to avoid reinfestation and prevent louse eggs from hatching. The lesson learned is that control of head lice in a community is dependent upon coordinated efforts to treat all infested individuals. Treating one child, or family group, in isolation risks only limited relief from head lice due to rapid reinfestation.

Based on our findings it may be appropriate to rethink the strategy for management of head louse infestation in Western Europe. Co-ordinated campaigns have been organised in some communities in the UK, Belgium, and Denmark using combing treatments [10], but the overall success rate can be low unless there is a considerable support network for families dealing with the problem. Even in Australia, where community based programmes have operated since the 1980s [11,12] the prevalence of head louse infestation remains high [12], probably because people use products affected by insecticide resistance, ineffective combing procedures, or do not have adequate professional support. Of course, it is also necessary for all families in the community to participate fully otherwise there is a risk for reinfestation. Any new strategy requires robust health promotion to communicate the need for full participation of the community, as in a programme conducted in the Isle of Man [13] where concerted efforts to identify all cases during a campaign resulted in reduced levels of infestation over the next 3-4 years. Similar effects were seen in the early use of malathion treatments in the late 1960s where there was a high degree of participation managed by local health services [14-16]. These managed programmes were so successful that the children were not reinfested for several weeks after treatment, which led investigators to draw the incorrect conclusion that malathion was capable of developing a residual effect on hair [14]. If it were possible to cover a similarly high proportion of the population at risk using reliably effective physically acting treatments like dimeticone lotions we anticipate the overall burden of infestation in many communities could be considerably reduced.

## Conclusion

In a community based, randomised, assessor blinded clinical trial we were unable to detect a difference in efficacy between dimeticone 4% lotion and dimeticone 4% lotion with nerolidol 2% added. We also found few cases with newly hatched nymphs after the first application of either treatment, indicating activity of the products to inhibit hatching of louse eggs. Dimeticone 4% lotion has been shown effective to cure head louse infestation in about 70-75% of patients in randomised trials in the UK but treating individuals in a community where prevalence of head lice and intensity of infestation is high often increases the risk of apparent treatment failure through reinfestation. Past studies have indicated that community treatment programmes of ectoparasites and endoparasites in Turkish villages have a high treatment success rate with few cases of reinfestation. In this study we also found a low level of reinfestation, probably due to the lower intensity of infestation than in the UK and the high level of participation in the study by families in the communities involved.

## Competing interests

IFB is a consultant to Thornton & Ross Ltd and to various other makers of pharmaceutical products, alternative therapies, and combs for treating louse infestations. None of the other authors is aware of having any competing interests.

## Authors' contributions

Conceived and designed the study: IFB, with input from OK and YO. Obtained ethics committee, health board, and education board approvals: OK, ICB, MTS, CB, MEL, NG, HK, YO. Performed the clinical investigations OK, ICB, HM, OE, MTS, CB, MEL, NG, HK, TT. Analysed the data: IFB. Wrote the paper: IFB, OK, ICB, YO, with comments and observations from all other authors.

## Pre-publication history

The pre-publication history for this paper can be accessed here:

http://www.biomedcentral.com/1471-2458/9/441/prepub
